# Fasting triglycerides and glucose index: a useful screening test for assessing insulin resistance in patients diagnosed with rheumatoid arthritis and systemic lupus erythematosus

**DOI:** 10.1186/s13098-019-0495-x

**Published:** 2019-11-27

**Authors:** Betsabe Contreras-Haro, Sandra Ofelia Hernandez-Gonzalez, Laura Gonzalez-Lopez, Maria Claudia Espinel-Bermudez, Leonel Garcia-Benavides, Edsaul Perez-Guerrero, Maria Luisa Vazquez-Villegas, Jose Antonio Robles-Cervantes, Mario Salazar-Paramo, Diana Mercedes Hernandez-Corona, Arnulfo Hernan Nava-Zavala, Jorge I. Gamez-Nava

**Affiliations:** 1 Departamento de Ciencias Biomédicas, Centro Universitario de Tonalá, Universidad de Guadalajara, Tonalá, Jalisco Mexico; 2 Unidad de Investigación Biomédica 02, and División de Investigación en Salud, Unidad Médica de Alta Especialidad Hospital de Especialidades, Centro Médico Nacional de Occidente, Instituto Mexicano del Seguro Social, Guadalajara, Jalisco Mexico; 3 Programa de Doctorado en Farmacología, Centro Universitario de Ciencias de la Salud, Universidad de Guadalajara, Guadalajara, Jalisco Mexico; 4 Programa de Doctorado en Salud Publica e Instituto de Investigación en Ciencias Biomédicas, Centro Universitario de Ciencias de la Salud, Universidad de Guadalajara (U de G), Guadalajara, Jalisco Mexico; 5 Division of Internal Medicine, Jalisco Institute of Reconstructive Surgery “Dr. José Guerrerosantos”, Guadalajara, Jalisco Mexico; 6 Programa Internacional, Facultad de Medicina, Universidad Autónoma de Guadalajara, Av. Patria No. 1201, Lomas del Valle, CP 45129 Zapopan, Jalisco Mexico; 7 Departamento de Inmunología y Reumatología, Hospital General de Occidente, Secretaria de Salud Jalisco, Zapopan, Jalisco Mexico

**Keywords:** Insulin resistance, Screening test, Connective tissue diseases

## Abstract

**Background:**

Insulin resistance (IR) is frequently observed in patients with rheumatoid arthritis (RA) or systemic lupus erythematosus (SLE). In clinical practice, IR assessment is limited to a low proportion of patients due to cost and equipment and technical expertise requirements. The surrogate index of triglycerides and glucose (TyG index) has been validated in non-rheumatic populations, showing adequate sensitivity and specificity for IR, although this index has not yet been used in connective tissue disorders. The aim of this study was to evaluate the frequency of insulin resistance (IR) using the validated surrogate index of triglycerides and glucose (TyG index) and to explore factors associated with IR in Mexican women with RA or SLE.

**Methods:**

Ninety-five female RA and 57 SLE patients were included in a cross-sectional study. Clinical and epidemiological variables were evaluated. IR was assessed using the TyG index with a cutoff value of > 4.68. Logistic regression analysis was performed to identify factors associated with IR excluding confounders.

**Results:**

IR frequency in the entire sample was 50%, higher than the 10% observed in non-rheumatic controls (p < 0.001). The frequency of IR was similar in SLE (49.1%) and RA (50.5%, p = 0.8) patients. IR was associated with a longer duration of hypertension and higher total cholesterol and low density lipoprotein cholesterol levels. Based on multivariate analysis, the duration of hypertension (OR: 1.06; 95% CI 1.002–1.12, p = 0.04), waist circumference (OR: 1.04; 95% CI 1.01–1.08, p = 0.007), uric acid levels (OR: 1.46; 95% CI 1.08–1.97, p = 0.01), RA (OR: 4.87; 95% CI 1.31–18.78, p = 0.01) and SLE (OR: 4.22; 95% CI 1.06–16.74, p = 0.04) were the main risk factors for IR.

**Conclusions:**

This study shows that the TyG index is a useful screening test for IR in RA and SLE patients. Future longitudinal studies should be performed with the aim of identifying the predictive value of TyG index results for identifying complications linked to IR.

## Background

Rheumatoid arthritis (RA) and systemic lupus erythematosus (SLE) are two types of systemic autoimmune diseases with a wide spectrum of severity. Both disorders are associated with an increased risk of cardiovascular disease (CVD) related to traditional risk factors such as hypertension, dyslipidaemia, smoking, and insulin resistance (IR), factors related to the inflammatory nature of these diseases and their treatments [1, 2]. These comorbidities are associated with higher mortality [3]. However, there is discordant information about the detection and management of comorbidities in RA patients between different countries [4]

IR can be defined as a decrease in the physiological homeostasis of glucose levels maintained by endogenous insulin [5]. Thus, IR is associated with increased insulin release and elevated blood concentrations of this hormone in an attempt to obtain an effective response to the glucose levels [5]. IR in the non-rheumatic population is a relevant risk factor for the development of type 2 diabetes mellitus (DM2), hypertension, and CVD [6–8]. In connective tissue disorders, the assessment of IR is relevant to prevent future complications, including CVD. Several publications have shown a wide variation in the frequency of IR in RA patients. These studies have demonstrated rates varying from 21.7 to 72.7% [1, 9–12]. In SLE, the prevalence of IR varies from 31.7 to 64.8% [12, 13]. However, despite the evidence reported by these studies regarding the high rates of IR in SLE and RA patients, the assessment of IR is infrequent in clinical practice. In the research studies, the most commonly used strategies to assess IR are the homeostatic model assessment of insulin resistance (HOMA-IR) and hyperinsulinemic-euglycemic clamp (Clamp).

Nevertheless, in the clinical scenario, these methods might have limitations due to decreased feasibility and high costs, especially in undeveloped countries. Thus, some authors have suggested the use of the fasting triglycerides and glucose TyG index as an alternative for the assessment of IR. The TyG index has been validated as a screening tool for IR in non-rheumatic populations. This surrogate index has been used as a screen for IR in diabetic and non-diabetic Mexican populations [14–16], and it has been implemented by researchers in several countries [17, 18]. Using HOMA-IR as the gold standard, the TyG index has a sensitivity of 84% and a positive predictive value of 81% for IR [16].

Moreover, using Clamp as the gold standard, the TyG index has a sensitivity of 96.5% and specificity of 85.0% for IR [15]. However, to date, there is no information regarding the use of the TyG index in patients suffering from rheumatic autoimmune diseases. Additionally, high variations in the prevalence of comorbidities and their management have been reported. Therefore, the present study aimed to evaluate the frequency of IR in Mexican women diagnosed with SLE or RA using the TyG index, and the second aim was to identify the factors most strongly associated with IR in these patients using multivariate models.

## Methods

This cross-sectional study was carried out from January 2016 to December 2018 and included consecutive patients attending a tertiary care centre in Guadalajara México recruited by invitation. Patients were eligible for inclusion in the study if they met the 1987 American College of Rheumatology (ACR) [19] criteria for RA or the 1982 ACR criteria for SLE [20]. Other criteria for eligibility were (a) age of 35 to 65 years, (b) body mass index (BMI) < 39.9 kg/m^2^, and (c) no participation in any research study. We excluded patients with antecedents of chronic renal failure (serum creatinine > 1.2 mg/dL), DM2, elevated transaminases (> 2-fold greater than the laboratory reference values), active infections (acute or chronic), cancer, or hypothyroidism. We also excluded pregnant or breastfeeding patients.

For comparison purposes, a control (CL) group comprising 50 female patients referred to the same hospital for studies of bone mineral density and/or patients seen for the assessment of diseases other than inflammatory rheumatic disorders was formed. The CL group was similar to the RA or SLE study group in terms of age (± 5 years) and BMI (± 2).

### Study protocol

Patients and CL participants were assessed by trained researchers using a standardized questionnaire that evaluated sociodemographic and epidemiological characteristics and comorbid diseases. Physical examination included weight and height, as well as waist and hip circumference. Body mass index (BMI; kg/m^2^) was used to classify patients into normal weight (18.5 to 24.9 kg/m^2^), overweight (25 to 29.9 kg/m^2^), and obese (≥ 30 kg/m^2^) groups. We assessed sedentary lifestyle, and sedentary patients were defined as patients who did not perform at least 150 min of moderate-intensity aerobic physical activity throughout the week, or an equivalent combination of moderate-and vigorous-intensity activity [21]. For patients with rheumatic autoimmune diseases, the questionnaire inquired about current pharmacologic treatment. Disease activity was evaluated in RA patients using the Disease Activity Score 28-joint counts (DAS28) [22]. This index reflects disease activity using a scale in which higher scores represent higher disease activity. In patients with SLE, disease activity was evaluated by the Systemic Lupus Erythematosus Activity Index (SLEDAI), which is a global disease activity index [23]. The SLEDAI assesses disease activity in the previous 10 days. Total SLEDAI scores range from a minimum of 0 to a maximum of 105. In the SLEDAI, higher scores represent higher disease activity.

### Fat mass measurement

Fat and lean mass were measured by dual-energy X-ray absorptiometry (DXA) using a Lunar 2000 Prodigy Advance DXA equipment (GE, Madison, WI, USA).

### Laboratory assessment

Blood samples were taken from all study participants after a 12-h fasting period and were placed in blood collection tubes (BD Vacutainer^**®**^). All blood samples were centrifuged and analysed on the day of clinical assessment. Analyses included glucose (mg/dL), total cholesterol (mg/dL), triglycerides (mg/dL), high-density lipoprotein cholesterol (HDL-c; mg/dL), low-density lipoprotein cholesterol (LDL-c; mg/dL) and serum uric acid (mg/dL) levels.

### Surrogate index of insulin resistance

Insulin resistance was assessed using the TyG index (based on triglyceride and fasting glucose values), as described by Simental-Mendia et al. [16]. The TyG index validation protocol has been described elsewhere [15], and the index score is calculated as follows (modified from [16]: TyG index = Ln [(TG × FG)/2]. In the formula, Ln denotes the natural logarithm, TG indicates triglyceride levels expressed in mg/dL, and FG is fasting glucose levels expressed in mg/dL.

The original authors of the TyG index validated this index in diabetic and other populations [17]. According to the authors, the best cutoff for diagnosing IR using the TyG index is > 4.68.

### Statistical analysis

Qualitative and quantitative variables are expressed as frequencies (%) and means ± standard deviations (SD), respectively. Comparisons of proportions between quantitative variables were performed using Chi-squared tests; comparisons of means between two groups were performed using Student’s *t*-tests, and comparisons between three groups were performed by one way analysis of variance (ANOVA) with T3-Dunnett’s post hoc analysis. We also examined whether these variables were associated with IR by analysing RA and SLE patients in each group separately.

Pearson’s correlation analysis was performed to identify the strength of the association between the TyG index score and the quantitative variables of the RA and SLE group and the CL group separately. Logistic regression models were computed to adjust for those variables that might influence the presence of IR in SLE and RA patients. Covariates were selected for inclusion in the different multivariate models based on two criteria: if they obtained a p ≤ 0.20 in the univariate analysis or if they had biologic plausibility for IR. Adjusted odds ratios (OR), as well as the corresponding 95% confidence intervals (95% CI), were estimated. Statistical significance was set at p ≤ 0.05.

## Results

The study sample comprised 152 patients diagnosed with rheumatic autoimmune disease, 95 of whom had RA and 57 of whom had SLE. IR was observed in 50% of these patients. A similar IR frequency was identified when the RA and SLE subgroups were analysed separately (50.5% vs. 49.1%, p = 0.8).

In Table 1, we show the comparison between the CL group and the RA and SLE group. In the comparison between the RA group and CL group, the RA group had a higher frequency of hypertension and menopause and a higher IR frequency. The RA group had a lower BMI. Regarding the comparison between the SLE group and the CL group, the SLE group was younger, had a higher frequency of hypertension, an elevated fat mass% and a higher IR frequency. Moreover, SLE patients were younger, had a lower frequency of menopause, and higher corticosteroid and chloroquine use than RA patients. No differences in disease duration were observed.

**Table 1:** Comparison of clinical characteristics between controls, rheumatoid arthritis (RA) and systemic lupus erythematosus (SLE)

Variables	Controls n = 50	RA n = 95	SLE n = 57	p
Age (years) mean ± SD	51.1 ± 7.7	53.9 ± 7.0	46.8 ± 9.8^**,a,b^	< *0.001*
Disease duration (years), mean ± SD	–	11.9 ± 9.1	12.1 ± 7.7	0.8
Smoking, n (%)	6 (12.0)	9 (9.9)	9 (15.8)	0.5
Sedentary lifestyle, n (%)	26 (52.0)	59 (62.1)	33 (57.9)	0.5
Hypertension, n (%)	4 (8.0)	32 (33.7)	16 (28.1)	0.003
Duration of hypertension (years), mean ± SD	7.3 ± 12.0	3.2 ± 6.8	2.0 ± 5.7	0.04
Menopause, n (%)	22 (45.8)	78 (82.1)	30 (52.6)	< *0.001*
Body mass index (kg/m^2^), mean ± SD	29.5 ± 2.3	28 ± 3.6*^,a^	29.1 ± 5.2	0.05
Underweight-Normal, n (%)	11 (22.0)	20 (21.1)	14 (24.6)	0.8
Overweight-obesity, n (%)	39 (78)	75 (78.9)	43 (75.4)	
Waist (cm), mean ± SD	92.4 ± 7.7	92 ± 9.5	92.7 ± 13.8	0.7
Total-cholesterol (mg/dL), mean ± SD	201.1 ± 33.5	196.6 ± 37.9	197.6 ± 40.9	0.7
Total-cholesterol (mmol/L), mean ± SD	3.2 ± 0.8	5 ± 0.9	5.1 ± 1.0	0.7
Elevated triglycerides, n (%)	29 (58.0)	25 (26.3)	24 (42.1)	0.07
HDL-cholesterol (mg/dL), mean ± SD	48.2 ± 14.3	52.3 ± 14.5	52.0 ± 14.6	0.2
HDL-cholesterol (mmol/L), mean ± SD	1.2 ± 0.3	1.3 ± 0.3	1.3 ± 0.3	0.2
LDL-cholesterol (mg/dL), mean ± SD	115.7 ± 30.0	114.9 ± 33.0	115.8 ± 31.0	0.9
LDL-cholesterol (mmol/L), mean ± SD	2.9 ± 0.7	2.9 ± 0.8	2.9 ± 0.8	0.9
Uric acid (mg/dL), mean ± SD	4.7 ± 0.9	4.4 ± 1.2	4.6 ± 1.1	0.1
Uric acid (mmol/L), mean ± SD	2.8 ± 0.5	2.6 ± 0.7	2.7 ± 0.6	0.1
TyG-index results				
Insulin Resistance, mean ± SD	3.83 ± 0.22	4.67 ± 0.21^**,a^	4.71 ± 0.30^**,a^	< *0.001*
Insulin Resistance, n (%)	5 (10.0)	48 (50.5)	28 (49.1)	< *0.001*
Fat mass% (DXA results), mean ± SD	46.2 ± 3.8	46.9 ± 4.9	48.5 ± 4.8**^,a^	*0.02*
Treatments				
Corticosteroid, n (%)	–	74 (77.9)	54 (94.7)	*0.006*
Chloroquine, n (%)	–	16 (15.1)	26 (48.1)	**< ***0.001*
Methotrexate, n (%)	–	55 (58.5)	9 (15.8)	< *0.001*
Leflunomide, n (%)	–	27 (28.4)	5 (8.8)	*0.004*
Biologics, n (%)	–	6 (6.3)	13 (22.8)	< *0.001*

Italic values indicate significance of p value (p < 0.05)

*HDL-cholesterol* high density lipoprotein cholesterol, *LDL-cholesterol* low density lipoprotein cholesterol, *DXA* Dual-Energy X-Ray Absortiometry, comparisons of quantitative variables between the three groups were made with one-way ANOVA. *p* value represents the statistical differences in at least one of the groups. To identify differences between two groups a corrected T3-Dunett analysis was performed

* p ≤ 0.05, ** p < 0.001

^a^Significant p-value between RA or SLE vs. controls

^b^Significant p-value between RA vs. SLE; elevated triglycerides were defined ≥ 1.69 mmol/L; biologics included in the analysis: adalimumab, rituximab and infliximab

Table 2 shows the comparison of the clinical characteristics between RA patients with IR and without IR. Patients with IR had a higher duration of hypertension (p = 0.02), a larger waist circumference (p = 0.008), higher total cholesterol (p = 0.005) and LDL-c levels (p = 0.02) and a lower HDL-c level (p = 0.001). No differences were observed in years of disease duration or pharmacologic treatment. The groups also had a similar fat mass (32.1 ± 6.3 kg vs. 31 ± 7.5 kg, p = 0.4) These data are not shown in the tables.

**Table 2:** Comparison between patients with rheumatoid arthritis (RA) with abnormal TyG

Variables	RA Abnormal TyG Index n = 48	RA Normal TyG index n = 47	p
Age (years) mean ± SD	54.0 ± 6.8	53.8 ± 7.5	0.8
Disease duration (years), mean ± SD	10.8 ± 8.9	12.9 ± 9.2	0.2
Smoking, n (%)	6 (12.5)	3 (6.7)	0.4
Sedentary lifestyle, n (%)	33 (68.8)	26 (55.3)	0.2
Hypertension, n (%)	19 (39.6)	13 (27.7)	0.2
Duration of hypertension (years), mean ± SD	4.8 ± 8.5	1.7 ± 3.9	*0.02*
Menopause, n (%)	42 (87.5)	36 (76.6)	0.1
Body mass index (kg/m^2^), mean ± SD	28.4 ± 3.4	27.5 ± 3.7	0.2
Waist (cm), mean ± SD	94.1 ± 8.1	89.0 ± 10.2	*0.008*
Total-cholesterol (mg/dL), mean ± SD	207.2 ± 42.1	185.8 ± 29.7	*0.005*
Total-cholesterol (mmol/L), mean ± SD	5.3 ± 1.0	4.8 ± 0.7	*0.005*
HDL-cholesterol (mg/dL), mean ± SD	47.5 ± 13.9	57.1 ± 13.5	*0.001*
HDL-cholesterol (mmol/L), mean ± SD	1.2 ± 0.3	1.4 ± 0.3	*0.001*
LDL-cholesterol (mg/dL), mean ± SD	122.6 ± 36.6	107.3 ± 27.3	*0.02*
LDL-cholesterol (mmol/L), mean ± SD	3.1 ± 0.9	2.7 ± 0.7	*0.02*
Uric acid(mg/dL), mean ± SD	4.6 ± 1.1	4.1 ± 1.3	0.07
Uric acid(mmol/L), mean ± SD	2.7 ± 0.6	2.4 ± 0.7	0.07
Fat mass% (DXA results), mean ± SD	46.9 ± 4.6	46.8 ± 5.1	0.8
Treatments			
Corticosteroid, n (%)	34 (70.8)	40 (85.1)	0.1
Chloroquine, n (%)	7 (14.5)	7 (14.8)	0.09
Methotrexate, n (%)	25 (53.2)	30 (63.8)	0.2
Leflunomide, n (%)	14 (29.2)	13 (27.7)	1.0
Biologics, n (%)	4 (8.3)	2 (4.3)	0.4

Italic values indicate significance of p value (p < 0.05)

An abnormal TyG index (TyG index > 4.68) suggests insulin resistance; *HDL-cholesterol* high density lipoprotein-cholesterol, *LDL-cholesterol* low density lipoprotein-cholesterol, *DXA* dual-energy X-ray absortiometry, TyG values are expressed in mean and standard deviation SD; comparisons between proportions were calculated by Χ^2^ test or Fisher´s exact test; comparisons between means by unpaired Student-t test; biologics included in the analysis: adalimumab, rituximab, infliximab

Table 3 shows the comparison of clinical and laboratory characteristics between SLE patients with IR and without IR. Patients with SLE and IR had an elevated waist circumference (p = 0.02), high total cholesterol (p = 0.001), LDL-c (p = 0.05), and uric acid (p = 0.01) levels, a higher fat mass, % (p = 0.007) and greater fat mass (37.8 ± 8.3 kg vs. 31.7 ± 8.2 kg, p = 0.007) These data are not shown in the table. No differences were observed between these two groups with respect to the frequency of smoking, sedentary lifestyle, menopause, pharmacologic treatment, or disease duration.

**Table 3:** Comparison between patients wit Systemic Lupus Erythematosus with abnormal TyG index and normal TyG index

Variables	SLE Abnormal TyG Index n = 28	SLE Normal TyG index n = 29	p
Age (yrs) mean ± SD	47.1 ± 11	46.5 ± 8.6	0.8
Disease duration (years), mean ± SD	13.0 ± 7.7	11.2 ± 7.7	0.2
Smoking, n (%)	4 (14.3)	5 (17.2)	1.0
Sedentary lifestyle, n (%)	13 (46.4)	20 (69)	0.08
Hypertension, n (%)	10 (35.7)	6 (20.7)	0.2
Duration of hypertension (years), mean ± SD	3.0 ± 6.4	1.1 ± 4.8	0.06
Menopause, n (%)	15 (53.6)	15 (51.7)	0.8
Body mass index (kg/m^2^), mean ± SD	30.0 ± 5.1	28.1 ± 5.1	0.1
Waist (cm), mean ± SD	96.9 ± 12.8	88.7 ± 13.8	*0.02*
Total-cholesterol (mg/dL), mean ± SD	214.7 ± 42.9	181.0 ± 31.6	*0.001*
Total-cholesterol (mmol/L), mean ± SD	5.5 ± 1.1	4.6 ± 0.8	*0.001*
HDL-cholesterol (mg/dL), mean ± SD	49.8 ± 14.2	54.2 ± 14.9	0.2
HDL-cholesterol (mmol/L), mean ± SD	1.2 ± 0.3	1.4 ± 0.3	0.2
LDL-cholesterol (mg/dL), mean ± SD	123.9 ± 35.1	108.0 ± 24.6	*0.05*
LDL-cholesterol (mmol/L), mean ± SD	3.2 ± 0.9	2.7 ± 0.6	*0.05*
Uric Acid(mg/dL), mean ± SD	5.0 ± 1.2	4.3 ± 0.9	*0.01*
Uric Acid(mmol/L), mean ± SD	2.9 ± 0.7	2.5 ± 0.5	*0.01*
Fat mass% (DXA results), mean ± SD	50.2 ± 4.5	46.7 ± 4.4	*0.007*
Treatments			
Corticosteroid, n (%)	28 (100)	26 (89.6)	0.2
Chloroquine, n (%)	13 (46.8)	13 (44.8)	1.0
Methotrexate, n (%)	4 (14.3)	5 (17.2)	1.0
Leflunomide, n (%)	2 (7.1)	3 (10.3)	1.0
Biologics, n (%)	7 (25)	6 (20.7)	0.6

Italic values indicate significance of p value (p < 0.05)

An abnormal TyG index (TyG index > 4.68) suggests insulin resistance; *HDL-cholesterol* high density lipoprotein-cholesterol, *LDL-cholesterol* low density lipoprotein-cholesterol, *DXA* dual-energy X-ray absortiometry; TyG values are expressed in mean and standard deviation SD; comparisons between proportions were calculated by Χ^2^ test or Fisher´s exact test; comparisons between means by unpaired Student-t test; biologics included in the analysis: adalimumab, rituximab, infliximab

Table 4 shows correlations between the TyG index value and the examined clinical variables in the controls and patients with RA and SLE. In the controls, a higher TyG index score was correlated with age (p = 0.02), duration of hypertension (p = 0.05), waist circumference (p = 0.01), and total cholesterol (p = 0.003) and serum uric acid (p = 0.008) levels. On the other hand, a lower TyG index score had a negative correlation with HDL-c levels (p = 0.003). Furthermore, in the RA group, the TyG index score had a positive correlation with the duration of hypertension (p = 0.02), BMI (p = 0.02), waist circumference (p = 0.002), and total cholesterol (p = 0.001), LDL-c (p = 0.01) and uric acid (p = 0.01) levels. In addition, a negative correlation was observed with HDL-c levels (p < 0.001). In addition, in the SLE group, there was a positive correlation with the duration of hypertension (p = 0.04), BMI (p = 0.05), waist circumference (p = 0.02), fat mass% (p = 0.002), and total cholesterol (p < 0.001), LDL-c (p = 0.01) and uric acid (p < 0.001) levels.

**Table 4:** Correlation TyG index with quantitative variables in Controls, RA and SLE

Variables	Controls TyG n = 50		RA TyG n = 95		SLE TyG n = 57	
	r	p	r	p	r	p
Age, years	0.32	*0.02*	0.02	0.8	0.001	0.9
Disease duration, years	–	–	0.01	0.9	0.06	0.6
Duration of hypertension, years	0.51	*0.05*	0.2	*0.02*	0.27	*0.04*
Menopause, years	0.17	0.3	− 0.06	0.5	0.13	0.3
Body Mass Index, kg/m^2^	0.07	0.6	0.23	*0.02*	0.25	*0.05*
Waist circumference, cm	0.36	*0.01*	0.31	*0.002*	0.29	*0.02*
Fat mass, %	0.25	0.07	0.003	0.9	0.39	*0.002*
Total-cholesterol, mmol/L	0.41	*0.003*	0.33	*0.001*	0.79	*< 0.001*
HDL-cholesterol, mmol/L	− 0.41	*0.003*	− 0.41	*< 0.001*	− 0.04	0.7
LDL-cholesterol, mmol/L	− 0.09	0.4	0.25	*0.01*	0.3	*0.01*
Uric acid, mmol/L	0.37	*0.008*	0.24	*0.01*	0.4	*< 0.001*
Disease activity index	–	–	0.15	0.1	0.06	0.6

Italic values indicate significance of p value (p < 0.05)

Association between variables were calculated by Pearson`s correlation; *HDL-cholesterol* high density lipoprotein-cholesterol, *LDL-cholesterol* low density lipoprotein-cholesterol, Disease activity index: DAS-28 for RA group and SLEDAI for SLE group

Moreover, no correlation was observed between the TyG index and disease activity in RA (using DAS28) or SLE (using SLEDAI).

Table 5 shows the logistic regression analysis results aimed at identifying the factors most strongly associated with IR according to a TyG index > 4.68 in the group of patients with rheumatic autoimmune diseases (women diagnosed with either RA or SLE) and the CL group. In the final model, after adjusting for study group (RA, SLE and CL), age, duration of hypertension, waist circumference, uric acid levels, only duration of hypertension (p = 0.04), waist circumference (p = 0.007), uric acid levels (p = 0.01), RA group (p = 0.01), and SLE group (p = 0.04) were associated with an increased risk of IR.

**Table 5:** Multivariate factors associated with the presence of abnormal TyG index > 4.68 in RA and SLE

Variables	OR	95% CI	p
Method forward stepwise			
Rheumatoid arthritis group	4.87	1.31–18.78	*0.01*
Systemic lupus erythematosus group	4.22	1.06–16.74	*0.04*
Age, years	Not in the model		
Hypertension, years	1.06	1.002–1.12	*0.04*
Waist circumference, cm	1.04	1.01–1.08	*0.007*
Uric acid, mmol/L	1.46	1.08–1.97	*0.01*

Multivariate analysis was performed by logistic regression. Model adjusted by age, hypertension, waist circumference, uric acid, RA, SLE and CL group (as reference)

Italic values indicate significance of p value (p < 0.05)

*OR* odds ratio, *95% CI* confidence interval

## Discussion

In the present study, approximately half of the women with RA or SLE had IR according to the TyG index. A finding of IR in these patients was associated with higher waist circumference, waist-hip ratio, and total cholesterol, LDL-c, and uric acid levels. In the multivariate analysis, after excluding other confounders, increased waist circumference, higher uric acid levels, a longer duration of hypertension, RA and SLE remained as risk factors associated with IR.

To the best of our knowledge, this is the first study using the TyG index for screening IR in SLE and RA patients. The TyG index is a useful tool for screening IR easy to perform and thus feasible for use in clinical settings. The TyG index results had adequate concordance with other standardized tests, such as HOMA-IR and Clamp [15, 16]. Thus, the TyG index has utility for clinicians and researchers as a screening tool for IR where using HOMA-IR is impractical or prohibitively expensive.

In patients with a systemic rheumatic disorder, it is essential for clinicians to discover IR early because it might modify therapeutic behaviours. Early detection of IR, for example, might lead physicians to avoid certain drugs that might increase IR, perform close surveillance or taper drug doses of corticosteroids. Additionally, the inclusion of antimalarials in therapy might offer benefits to IR patients. Antimalarials have well-known favourable effects on lipid profiles and glucose levels. Therefore, the introduction of these therapeutic and preventive measures might decrease the risk of CVD or DM2 development associated with IR [24]. To date, CVD remains the leading cause of mortality in patients affected by rheumatic inflammatory disorders, particularly SLE and RA [25, 26]. IR frequency is different in different countries, and this phenomenon has been observed in various studies of RA and SLE [1, 7–11]. Table 6 indicates the wide variability in the frequency of IR across different studies.

**Table 6:** Frequency of Insulin Resistance in different studies and associations with clinical variables

Author, year	Country	Study design	Method of Assessment for IR	Study groups		Frequency of IR		Results of univariate analysis or correlations between IR and variables in RA and or SLE	Variables associated to IR in RA and or SLE in the multivariate analysis
Shahin et al., 2010 [10]	Egypt	Cross-sectional	HOMA-IR	RA CL	n = 66 n = 40	RA CL	72.7% NA	IR correlated with: body mass index, total cholesterol, triglycerides, LDL-cholesterol, insulin, glucose, erythrocyte sedimentation rate, IR not correlated with: HDL-c	Multiple linear logistic regression: insulin, HDL-c, total-cholesterol, erythrocyte sedimentation rate
Manrique et al., 2016 [1]	Spain	Cohort	HOMA-IR	RA CL	n = 46 n = 45	RA CL	21.7% 15.6%	IR correlated with: waist-hip ratio, total fat mass, disease duration, rheumatoid factor, TNF-IR not correlated with: age, body mass index, waist circumference, total free fat mass, blood pressure, HDL-c, LDL-c, total-cholesterol, triglycerides	Box-Cox: total fat mass, disease duration
Costa et al., 2016 [9]	Brazil	Cross-sectional	HOMA-IR	RA CL	n = 173 n = 97	RA CL	47% NA	IR associated with: DAS 28, C-reactive protein, erythrocyte sedimentation rate, IR not associated with: disease duration, RA pharmacologic treatment	ND
Müller et al., 2017 [11]	Estonia	Cross-sectional	HOMA-IR	RA CL	n = 92 n = 321	RA CL	48.5% 22.5%	IR associated with: disease duration, DAS 28, C-reactive protein, TNF-a, IL-6 IR not associated with: age, body mass index, rheumatoid factor, glucocorticosteroid use, disease modifying drugs use, total fat mass	Binomial logistic regression: appendicular lean mass and fat mass index by DEXA, high DAS 28 score
Lozovoy et al., 2013 [13]	Brazil	Cross-sectional	HOMA-IR	SLE CL	n = 111 n = 125	SLE CL	64.8% NA	IR associated with: body mass index, gamma-glutamyl transferase, antihypertensive drugs use, IR not associated with: age, smoking, disease duration, SLEDAI, SLE pharmacologic treatment	ND
Gazareen et al., 2014 [12]	Egypt	Cross-sectional	HOMA-IR	SLE RA CL	n = 35 n = 35 n = 20	SLE RA CL	37.1% 54.2% NA	IR in SLE correlated with: triglycerides, erythrocyte sedimentation rate, C-reactive protein, disease activity (SLAM), IR in SLE not correlated with: body mass index, total cholesterol, HDL-c, LDL-c, IR in RA correlated with: total cholesterol, triglycerides, LDL-c, erythrocyte sedimentation rate, C-reactive protein, DAS 28, IR in RA not correlated with: age, body mass index, disease duration, HDL-c	ND
Present study, 2019	Mexico	Cross-sectional	TyG index	SLE RA CL	n = 66 n = 107 n = 50	SLE RA CL	54.8% 55.1% 10%	IR in SLE correlated with: duration of hypertension, body mass index, waist circumference, fat mass, total-cholesterol, LDL-c, uric acid, IR in SLE not correlated with: age, HDL-c, disease duration, SLEDAI, IR in RA correlated with: duration of hypertension, body mass index, waist circumference, total cholesterol, HDL-c, LDL-c, uric acid, IR in RA not correlated with: age, fat mass, disease duration, DAS 28	Logistic regression: duration of hypertension, waist circumference and uric acid

*HOMA-IR* homeostatic model assessment for insulin resistance, *TyG index* triglyceride glucose-index, *RA* rheumatoid arthritis, *SLE* systemic lupus erythematosus, *CL* control group, *LDL-c* low density lipoprotein cholesterol, *HDL-c* high density lipoprotein cholesterol, *TNF-a* tumor necrosis factor-α, *IL-6* interleukin 6, *DAS 28* Disease Activity Score for RA, *SLAM* systemic lupus activity measure, *ND* not done

In the present study, IR was noted in 50.5% of the patients with RA. However, as this is the first report using the TyG index for identifying IR, our findings can only be compared to those that used HOMA-IR to detect IR. The highest prevalence of IR in RA patients (72.7%) has been observed in Egypt [10]. Our results are similar to those reported for the RA population in Brazil, where the frequency of IR in RA patients was reported to be 47% [9].

In relation to the factors associated with IR, our findings are supported by the results published by Shahin et al. who noted a positive correlation between IR and BMI and a negative correlation between IR and HDL-c levels [10].

We did not find an association between an abnormal TyG index and SLE and RA disease activity. This finding is not in agreement with previously published studies of RA [9–12]. However, regarding SLE, there are disagreements between studies; while some authors observed an association between disease activity and IR [12], other authors did not find such an association [13]. Various studies have reported an increase in IR in association with disease activity assessed by the DAS28 in RA patients [7, 8, 10]. We identified several differences in the clinical characteristics of patients included in the studies and observed a relationship between disease activity and IR in RA. While the studies performed by Manrique et al. [1] and Müller et al. [11], which included RA patients with a shorter disease duration (< 1 year), we included RA patients with a mean disease duration of 11 years. This important difference influenced the results because patients with a long disease duration have many other factors that can decrease the effect of disease activity on IR. In Brazil, Costa et al. used HOMA-IR and observed an association between IR and DAS28 scores [7].

In SLE patients, we did not observe a relationship. Gazareen et al. observed a relationship between IR and disease activity using HOMA-IR [12]. Nevertheless, these authors utilized the systemic lupus activity measure (SLAM) for the assessment of disease activity. Instead, we used a different disease activity index; our patients were assessed by the SLEDAI. There are several differences between the SLEDAI and SLAM [27].The SLAM evaluates disease activity in the previous month, whereas the SLEDAI evaluates disease activity in the previous 10 days. The SLEDAI is easily applicable and includes more objective measures of disease activity. Instead, the SLAM includes some subjective items assessed by the patients [27].

In accordance with the findings of our study, Lozovoy et al. did not identify an association between disease activity assessed by the SLEDAI and IR [13]. The SLE patients had similar disease durations as those of the patients included in our study. Finally, we must consider differences in other confounder variables, such as overweight and obesity, that were highly prevalent in the patients in our study and might influence the lack of association with disease activity.

A limitation of our study was that most of our RA and SLE patients had a long disease duration, making it difficult to compare the results with studies of patients with short disease durations. Future follow-up studies should assess the increase in the rate of IR throughout the evolution of disease.

On the other hand, Manrique et al. examined the prevalence of IR in untreated RA Spanish patients, 21.7% of whom were found to be insulin resistant [1]. Manrique et al. found an association between IR and the symptom duration of RA, a finding that was not observed by us. Our data indicate that IR can be observed at any point in the disease course; therefore, the TyG index should be considered in patients where the assessment of IR should be performed periodically.

A diagnosis of IR was obtained in 49.1% of our patients with SLE, which is below the 64.8% reported for Brazilian patients with SLE by Lozovoy et al. who noted an association between IR and BMI [13]. We observed a non-significant trend of higher BMI in our patients with RA or SLE with IR. Nevertheless, insufficient statistical power for detecting these differences in our study cannot be excluded. Gazareen et al. examined IR in SLE and RA patients [12]. These authors noticed a lower IR prevalence in SLE patients compared with RA patients. However, we did not observe differences in the frequency of IR between RA and SLE patients because our patients with SLE were significantly younger than our RA patients. This suggests that clinicians should assess IR in patients with autoimmune rheumatic disorders at any age or at any point in the disease course.

In contrast to the findings observed by other authors [1, 8, 9], we found, based on the adjusted analysis, that IR was associated with higher waist circumference, higher uric acid levels and a longer duration of hypertension in both the RA and SLE groups.

Race as well as a wide range of environmental factors, might contribute to the differences observed in the IR frequency among non-rheumatic populations [28, 29]. We can assume, based on the data shown in Table 6, that the influence of race/ethnicity on the presence of IR also prevails in RA and SLE. HOMA-IR is a widely used measure to detect IR, but different cutoff values have been used across studies. Moreover, at present, there is no consensus on the HOMA-IR cutoff value for RA and SLE patients; standard cutoff points are followed, but these may vary due to differences among ethnic groups and clinical settings [11]. The TyG index also provides a valid cutoff point for the Mexican population, although we recommend evaluating the cutoff point when this index is applied to other races and ethnicities.

In the present study, IR was related to higher waist circumference, higher uric acid levels and a longer duration of hypertension, being waist circumference a major determinant than inflammation in AR and SLE patients [30–32]. Waist circumference is associated with abdominal obesity, increased levels of free fatty acids [33], saturated fatty acids (SFA), modified low-density lipoproteins (LDLs) and diacylglycerol (DAG) [34]. These lipids and lipopolysaccharides (LPS) contribute to the activation of Toll-like receptor 2 (TLR2), Toll-like receptor 4 (TLR4) and protein kinase C (PKC) [35]. TLR2/4 are expressed by macrophages, adipocytes, skeletal muscle, and pancreatic B cells and in the brain [34, 35]. TLR2/4 activation causes a proinflammatory response in autoimmune diseases and contributes to an obesity-induced inflammatory response by increasing TNF-a and IL-6 levels in RA and TNF-a, IL-6, IL-23, and IL-10 levels in SLE [7]. Some inflammatory mediators of the TLR2/4 pathway, including JNK, IL-1, and IL-6, induce IR by impairing insulin receptor substrates and PI3K–AKT pathway activation [34, 36]. Additionally, an increase in free fatty acids, which is observed in IR, decreases the secretion of adiponectin, an anti-inflammatory adipokine that increases the secretion of and sensitivity to insulin. Adiponectin impairs the LPS-induced activation of TLR4 signalling pathways [35], and low levels of this adipokine have been associated with IR [35, 37, 38]. Moreover, DAG PKC activation contributes to lipid-induced hepatic insulin resistance through phosphorylation and the consequent inhibition of the insulin receptor [39].

Elevated uric acid levels are known to contribute to IR. A possible mechanism is that higher uric acid levels induce oxidative stress by increasing tissue NADPH oxidase levels and reactive oxygen species (ROS) production, which cause oxidative damage in insulin signalling cells [40]. Some other authors have also proposed that elevated uric acid levels are related to renal absorption of urates contributing to the perpetuation of the cycle of hyperuricaemia [41]. Hyperuricaemia has been proposed as the greatest risk factor for hypertension, but the mechanisms remain uncertain. It may be elicited by the induction of renal arteriolopathy through vascular smooth cell proliferation or the increased expression of epithelial sodium channel units, causing a decrease in urinary sodium excretion [42, 43]. An increased frequency of hyperuricaemia has been described in RA and SLE [44]. On the other hand, insulin has physiologic effects, induces vasorelaxation by stimulating nitric oxide production in the endothelium, and regulates sodium homeostasis by enhancing sodium reabsorption in the kidneys, which contributes to blood pressure regulation [45, 46].

At present, Clamp is considered the gold standard for IR diagnosis [47]. In this technique, the plasma insulin concentration is elevated and maintained by a continuous infusion of insulin and concomitant dextrose infusion to achieve euglycemia (a glucose concentration similar to basal levels). The glucose infusion rate equals glucose uptake by all tissues and is therefore a measure of tissue sensitivity to exogenous insulin [48].

However, this test is not easily available in clinical settings. The major limitations of this test include the cost of the test and the equipment and technical training requirements [44]. Consequently, Clamp has limited efficiency as a screening test for epidemiological studies. Instead, HOMA-IR is typically used to measure IR, as it is more affordable and easier to perform. As HOMA-IR is not always available in all clinical settings in developing countries and because it is not easy to perform repeated measures of HOMA-IR in wide populations, it is essential to find a suitable alternative [49]. In this study, we propose using the TyG index as a suitable alternative, as it is a feasible low-cost method for screening IR in high-risk populations, including patients with chronic autoimmune disorders, such as RA and SLE. The TyG index can be used as substitute for other tests in clinical settings where these tests are not feasible, and it has been demonstrated that the TyG index has high sensitivity and specificity compared with HOMA-IR and Clamp [15, 16].

Our study has several limitations. One of the major limitations is derived from the cross-sectional design, as this yields only a snapshot at a particular point in time. Thus, further longitudinal studies are needed to identify IR incidence and clarify the role of the risk factors reported here in the cumulative incidence of IR and its complications. On the other hand, in the present study, we used a surrogate index for IR that was not compared with a gold standard such as Clamp or HOMA-IR. Nevertheless, the TyG index has been widely validated in non-rheumatic populations and has been proposed as a screening tool for IR because of its good sensitivity and specificity when compared with Clamp or HOMA-IR [15, 16]. Future studies should compare these 3 methods in rheumatic populations. Another important limitation of the present study was that the patients with RA and SLE were significantly different in age and menopause status. This reflects the fact that SLE is observed in younger patients than RA. Although these differences were observed in the univariate analysis, the frequency of IR according to the TyG index was similar, leading to the hypothesis that SLE patients might develop abnormalities of insulin resistance earlier than RA patients. Another limitation is that most of the patients included in the study had a long disease duration. This was expected since we chose to use the 1987 ACR criteria for RA. The 1987 ACR criteria are more specific than the 2010 ACR/EULAR criteria but have a low sensitivity for early RA [50]. The findings of this study are therefore limited to SLE or RA patients with long disease durations; because we only had 6 RA patients and 5 SLE patients with a disease duration < 2 years, we did not have sufficient statistical power to compare the prevalence of IR in patients earlier in the disease course. Future studies should use a larger sample size of patients with short and long disease durations to make comparisons possible. Finally, we did not find any association between IR and treatments, including glucocorticoids, synthetic disease controlling anti-rheumatic drugs or biologic agents. Nevertheless, because this was a cross-sectional study, we could not study changes in IR over time due to these drugs. Future cohort studies should address this issue.

We conclude that this study is the first to use the TyG index for the assessment of IR prevalence in SLE and RA patients. We found that approximately half of patients with these diseases met the criteria of the TyG index for IR. These interesting results show the ability of the TyG index to detect the biological variability of these biomarkers in RA and SLE patients. Thus, we suggest the use of the TyG index as an alternative to HOMA-IR or Clamp for identifying IR cases in epidemiological studies or in clinical consults where the measurement of IR can be applied as a screening tool, especially clinical settings where HOMA-IR and Clamp are not feasible. Early IR detection and follow-up designs that include both the TyG index as a screening tool and comparisons with HOMA-IR or Clamp should be required in SLE and RA patients to plan strategies for preventing the development of complications such as DM2 and CVD associated with IR.

## Conclusions

The TyG index is a feasible method for screening IR in patients with rheumatic diseases, as they are at high risk for the development of DM2 and CVD. The TyG index might be useful in planning strategies for preventing these complications. Waist circumference as a marker of overweight-obesity is a greater determinant than inflammation in insulin resistance in this type of patients, and should be taken into account by clinicians to emphasize weight control in order to avoid complications associated with unhealthy weight gain.

## Data Availability

The datasets used and/or analyzed during the current study are available from the corresponding author on a reasonable request.

## Abbreviations

ACR: American College of Rheumatology
BMI: body mass index
CI: confidence interval
CL: Control Group
Clamp: hyperinsulinemic-euglycemic Clamp
CVD: cardiovascular disease
DM2: type 2 diabetes mellitus
DAS28: Disease Activity Score 28-joint counts
DAG: diacylglycerol
DXA: dual-energy X-ray absorptiometry
FG: fasting glucose
HDL-c: high density lipoprotein cholesterol
HOMA-IR: homeostatic model assessment
IR: insulin resistance
LDL-c: low density lipoprotein cholesterol
OR: odds ratio
PI3k–AKT: phosphatidylinositol 3-kinase pathway
PKC: protein kinase C
RA: rheumatoid arthritis
SD: standard deviation
SFA: saturated fatty acids
SLAM: systemic lupus activity measure
SLE: systemic lupus erythematosus
SLEDAI: Systemic Lupus Erythematosus Activity Index
TLR2: toll like receptor 2
TLR4: toll like receptor 4
TNF-α: tumor necrosis factor α
TyG index: Triglyceride Glucose-Index
TG: triglycerides

## References

[1] Manrique-Arija S, Ureña I, Valdivielso P, Rioja J, Jiménez-Núñez FG, Irigoyen MV (2016). Insulin resistance and levels of adipokines in patients with untreated early rheumatoid arthritis. Clin Rheumatol.

[2] Erdozain JG, Ruiz Irastorza G (2015). Síndrome metabólico en pacientes con lupus eritematoso sistémico: causas y consecuencias. Med Clín.

[3] Ferguson LD, Siebert S, McInnes IB, Sattar N (2019). Cardiometabolic comorbidities in RA and PsA: lessons learned and future directions. Nat Rev Rheumatol.

[4] Dougados M, Soubrier M, Antunez A, Balint P, Balsa A, Buch MH (2014). Prevalence of comorbidities in rheumatoid arthritis and evaluation of their monitoring: results of an international, cross-sectional study (COMORA). Ann Rheum Dis.

[5] Nicolau J, Lequerré T, Bacquet H, Vittecoq O (2017). Rheumatoid arthritis, insulin resistance, and diabetes. Joint Bone Spine.

[6] Aviña-Zubieta JA, Choi HK, Sadatsafavi M, Etminan M, Esdaile JM, Lacaille D (2008). Risk of cardiovascular mortality in patients with rheumatoid arthritis: a meta-analysis of observational studies. Arthritis Rheum.

[7] Chen D-Y, Chen Y-M, Hsieh T-Y, Hsieh C-W, Lin C-C, Lan J-L (2015). Significant effects of biologic therapy on lipid profiles and insulin resistance in patients with rheumatoid arthritis. Arthritis Res Ther..

[8] Ormseth M, Swift L, Fazio S, Linton M, Raggi P, Solus J (2013). Free fatty acids are associated with metabolic syndrome and insulin resistance but not inflammation in systemic lupus erythematosus. Lupus..

[9] Costa NT, Veiga Iriyoda TM, Kallaur AP, Delongui F, Alfieri DF, Lozovoy MAB (2016). Influence of insulin resistance and TNF-α on the inflammatory process, oxidative stress, and disease activity in patients with rheumatoid arthritis. Oxid Med Cell Longev..

[10] Shahin D, Eltoraby E, Mesbah A, Houssen M (2010). Insulin resistance in early untreated rheumatoid arthritis patients. Clin Biochem.

[11] Müller R, Kull M, Lember M, Põlluste K, Valner A, Kallikorm R (2017). Insulin resistance in early rheumatoid arthritis is associated with low appendicular lean mass. Biomed Res Int.

[12] Gazareen S, Fayez D, El-Najjar M, Dawood A, Essa E, El-zorkany K (2014). Study of insulin resistance in patients with systemic lupus erythematosus and rheumatoid arthritis. Menoufia Med J..

[13] Lozovoy M, Simão A, Oliveira S, Iryioda T, Panis C, Cecchini R (2013). Relationship between iron metabolism, oxidative stress, and insulin resistance in patients with systemic lupus erythematosus. Scand J Rheumatol.

[14] Espinel-Bermúdez MC, Robles-Cervantes JA, del Sagrario Villarreal-Hernández L, Villaseñor-Romero JP, Hernández-González SO, González-Ortiz M (2015). Insulin resistance in adult primary care patients with a Surrogate Index, Guadalajara, 2012. Mexico. J Investig Med..

[15] Guerrero-Romero F, Simental-Mendía LE, González-Ortiz M, Martínez-Abundis E, Ramos-Zavala MG, Hernández-González SO (2010). the product of triglycerides and glucose, a simple measure of insulin sensitivity. Comparison with the euglycemic-hyperinsulinemic clamp. J Clin Endocrinol Metab..

[16] Simental-Mendía LE, Rodríguez-Morán M, Guerrero-Romero F (2008). The product of fasting glucose and triglycerides as surrogate for identifying insulin resistance in apparently healthy subjects. Metab Syndr Relat Disord..

[17] Navarro-González D, Sánchez-Íñigo L, Pastrana-Delgado J, Fernández-Montero A, Martinez JA (2016). Triglyceride–glucose index (TyG index) in comparison with fasting plasma glucose improved diabetes prediction in patients with normal fasting glucose: the Vascular-Metabolic CUN cohort. Prev Med.

[18] Yu X, Wang L, Zhang W, Ming J, Jia A, Xu S (2019). Fasting triglycerides and glucose index is more suitable for the identification of metabolically unhealthy individuals in the Chinese adult population: a nationwide study. J Diabetes Investig..

[19] Arnett FC, Edworthy SM, Bloch DA, Mcshane DJ, Fries JF, Cooper NS (1988). The american rheumatism association 1987 revised criteria for the classification of rheumatoid arthritis. Arthr Rheum.

[20] Tan EM, Cohen AS, Fries JF, Masi AT, Mcshane DJ, Rothfield NF (1982). The 1982 revised criteria for the classification of systemic lupus erythematosus: REVISED CRITERIA FOR SLE. Arthr Rheum.

[21] World Health Organization. Global recommendations on physical activity for health; 2010. https://www.who.int/dietphysicalactivity/factsheet_recommendations/en/. Accessed 2019 Oct 19. 26180873

[22] Prevoo MLL, Hof MA, Kuper HH, Van Leeuwen MA, Van De Putte LBA, Van Riel PLCM (1995). Modified disease activity scores that include twenty-eight-joint counts. Development and validation in a prospective longitudinal study of patients with rheumatoid arthritis. Arthr Rheum..

[23] Bombardier C, Gladman DD, Urowitz MB, Caron D, Chang CH, Austin A (1992). Derivation of the sledai. A disease activity index for lupus patients. Arthr Rheum.

[24] Reaven G (2012). Insulin resistance and coronary heart disease in nondiabetic individuals. Arterioscler Thromb Vasc Biol.

[25] Knight JS, Kaplan MJ (2013). Cardiovascular disease in lupus: insights and updates. Curr Opin Rheumatol.

[26] Zegkos T, Kitas G, Dimitroulas T (2016). Cardiovascular risk in rheumatoid arthritis: assessment, management and next steps. Ther Adv Musculoskelet Dis..

[27] Lam GKW, Petri M (2005). Assessment of systemic lupus erythematosus. Clin Exp Rheumatol.

[28] Hasson BR, Apovian C, Istfan N (2015). Racial/ethnic differences in insulin resistance and beta cell function: relationship to racial disparities in type 2 diabetes among African Americans versus caucasians. Curr Obes Rep..

[29] Spanakis EK, Golden SH (2013). Race/ethnic difference in diabetes and diabetic complications. Curr Diab Rep..

[30] Zadeh-Vakili A, Tehrani FR, Hosseinpanah F (2011). Waist circumference and insulin resistance: a community based cross sectional study on reproductive aged Iranian women. Diabetol Metab Syndr..

[31] Pourfarzam M, Zadhoush F, Sadeghi M (2016). The difference in correlation between insulin resistance index and chronic inflammation in type 2 diabetes with and without metabolic syndrome. Adv Biomed Res..

[32] Cohen JI, Maayan L, Convit A (2012). Preliminary evidence for obesity-associated insulin resistance in adolescents without elevations of inflammatory cytokines. Diabetol Metab Syndr..

[33] Patel P, Abate N (2013). Body fat distribution and insulin resistance. Nutrients..

[34] Jialal I, Kaur H, Devaraj S (2014). Toll-like receptor status in obesity and metabolic syndrome: a translational perspective. J Clin Endocrinol Metab.

[35] Kim JJ, Sears DD (2010). TLR4 and insulin resistance. Gastroenterol Res Pract.

[36] Ruscitti P, Ursini F, Cipriani P, Greco M, Alvaro S, Vasiliki L (2019). IL-1 inhibition improves insulin resistance and adipokines in rheumatoid arthritis patients with comorbid type 2 diabetes: an observational study. Medicine (Baltimore)..

[37] Makki K, Froguel P, Wolowczuk I (2013). Adipose tissue in obesity-related inflammation and insulin resistance: cells, cytokines, and chemokines. ISRN Inflamm..

[38] Tabata S, Yoshimitsu S, Hamachi T, Abe H, Ohnaka K, Kono S (2009). Waist circumference and insulin resistance: a cross-sectional study of Japanese men. BMC Endocr Disord..

[39] Gassaway BM, Petersen MC, Surovtseva YV, Barber KW, Sheetz JB, Aerni HR (2018). PKCε contributes to lipid-induced insulin resistance through cross talk with p70S6 K and through previously unknown regulators of insulin signaling. Proc Natl Acad Sci.

[40] Zhu Y, Hu Y, Huang T, Zhang Y, Li Z, Luo C (2014). High uric acid directly inhibits insulin signalling and induces insulin resistance. Biochem Biophys Res Commun.

[41] Robles-Cervantes JA, Ramos-Zavala MG, González-Ortiz M, Martínez-Abundis E, Valencia-Sandoval C, Torres-Chávez A (2011). Relationship between serum concentration of uric acid and insulin secretion among adults with type 2 diabetes mellitus. Int J Endocrinol..

[42] Mazzali M, Kanellis J, Han L, Feng L, Xia Y-Y, Chen Q (2002). Hyperuricemia induces a primary renal arteriolopathy in rats by a blood pressure-independent mechanism. Am J Physiol-Ren Physiol..

[43] Xu W, Huang Y, Li L, Sun Z, Shen Y, Xing J (2016). Hyperuricemia induces hypertension through activation of renal epithelial sodium channel (ENaC). Metabolism..

[44] Chandrashekara S, Dhote SV, Anupama KR (2019). The differential influence of immunological process of autoimmune disease on lipid metabolism: a study on RA and SLE. Indian J Clin Biochem.

[45] Horita S, Seki G, Yamada H, Suzuki M, Koike K, Fujita T (2011). Insulin resistance, obesity, hypertension, and renal sodium transport. Int J Hypertens..

[46] Manhiani MM, Cormican MT, Brands MW (2011). Chronic sodium-retaining action of insulin in diabetic dogs. Am J Physiol-Ren Physiol..

[47] Pisprasert V, Ingram KH, Lopez-Davila MF, Munoz AJ, Garvey WT (2013). Limitations in the use of indices using glucose and insulin levels to predict insulin sensitivity: impact of race and gender and superiority of the indices derived from oral glucose tolerance test in African Americans. Diabetes Care.

[48] DeFronzo RA, Tobin JD, Andres R (1979). Glucose clamp technique: a method for quantifying insulin secretion and resistance. Am J Physiol.

[49] Gutch M, Kumar S, Razi S, Gupta K, Gupta A (2015). Assessment of insulin sensitivity/resistance. Indian J Endocrinol Metab..

[50] van der Linden MPM, Knevel R, Huizinga TWJ, van der Helm-van Mil AHM (2011). Classification of rheumatoid arthritis: comparison of the 1987 American College of Rheumatology criteria and the 2010 American College of Rheumatology/European League Against Rheumatism criteria. Arthritis Rheum.

